# Connexin43 in Germ Cells Seems to Be Dispensable for Murine Spermatogenesis

**DOI:** 10.3390/ijms22157924

**Published:** 2021-07-25

**Authors:** Kristina Rode, Marion Langeheine, Bettina Seeger, Ralph Brehm

**Affiliations:** 1 Institute of Anatomy, University of Veterinary Medicine Foundation, 30173 Hanover, Germany; kristina.rode@tiho-hannover.de (K.R.); marion.langeheine@tiho-hannover.de (M.L.); 2 Institute for Food Quality and Food Safety, University of Veterinary Medicine Foundation, 30173 Hanover, Germany; Bettina.seeger@tiho-hannover.de

**Keywords:** Connexin43, spermatogenesis, testis, male germ cells, Sertoli cell, gap junction, intercellular communication

## Abstract

Testicular Connexin43 (Cx43) connects adjacent Sertoli cells (SC) and SC to germ cells (GC) in the seminiferous epithelium and plays a crucial role in spermatogenesis. However, the distinction whether this results from impaired inter-SC communication or between GC and SC is not possible, so far. Thus, the question arises, whether a GC-specific Cx43 KO has similar effects on spermatogenesis as it is general or SC-specific KO. Using the Cre/loxP recombinase system, two conditional KO mouse lines lacking Cx43 in premeiotic (pGCCx43KO) or meiotic GC (mGCCx43KO) were generated. It was demonstrated by qRT-PCR that Cx43 mRNA was significantly decreased in adult pGCCx43KO mice, while it was also reduced in mGCCx43KO mice, yet not statistically significant. Body and testis weights, testicular histology, tubular diameter, numbers of intratubular cells and Cx43 protein synthesis and localization did not show any significant differences in semi-quantitative Western blot analysis and immunohistochemistry comparing adult male KO and WT mice of both mouse lines. Male KO mice were fertile. These results indicate that Cx43 in spermatogonia/spermatids does not seem to be essential for successful termination of spermatogenesis and fertility as it is known for Cx43 in somatic SC, but SC-GC communication might rather occur via heterotypic GJ channels.

## 1. Introduction

Spermatogenesis, as a highly regulated process of mitosis and meiosis, requires intensive regulation to synchronize processes of germ cell (GC) proliferation, migration and differentiation. Besides endocrine, paracrine and autocrine regulation, intercellular communication via gap junctions (GJ) is essential for the completion of normal spermatogenesis [1,2]. GJs are intercellular plasma membrane channels allowing for direct electric and metabolic coupling of the connected cells. GJ-mediated communication can occur between cells of the same cell type (homocellular) or between different cell types (heterocellular). The constituting proteins of GJ channels are connexins (Cxs). Cxs are composed of four transmembrane domains, two extracellular loops, one cytoplasmic loop and the cytoplasmic N- and C-terminus. Forming a GJ channel between two cells, each participating cell contributes one hemichannel (=connexon) at opposing plasma membranes, which in turn consists of six Cx [3,4,5]. Besides classical GJ intercellular communication (GJIC), GJs are also able to provide GJ mediated cell adhesion independent from their channel function, which is critical for cell migration [6,7,8].

In mice, the expression of at least 19 different Cx genes could be identified [9]. In testicular GC of rats, transcripts of Cx26, -31, -32, -33, -37, -40, -43, -45 and -50 have been identified [10]. In mammalian testes, the predominant Cx is Cx43, which can be found between Leydig cells, peritubular cells, SC and GC, namely spermatogonia, spermatocytes and spermatids [10,11,12,13,14,15,16,17,18,19]. So far, the knowledge about the specific roles of Cx43 in GC is very limited. Several authors have shown that, besides in SC-SC communicating junctions, Cx43 is also present in different GC populations (spermatogonia, spermatocytes and spermatids) and that it forms functional GJ between those GC and SC [10,11,12,13,14,16,20,21,22,23]. Among themselves, GCs communicate by intercellular bridges due to incomplete cytokinesis [24,25], which allows the transmission of substances from SC via GJ to an entire GC clone [16].

To investigate the role of Cx43 within the testis, different animal models were developed. In mice showing a general knockout (KO) of Cx43, the offspring were not viable and died at birth due to cardiopulmonary malformations [26]. Investigations of fetal testes of those KO mice revealed reduced testicular weight and a decrease in GC number as compared to wild type (WT) control testes. The authors suggested that Cx43 GJ might be determinant for the migration, proliferation and/or survival of primordial GC [27]. Accordingly, Francis and Lo (2006) could show that primordial GCs are GJ communication competent cells with dye coupling being markedly reduced in Cx43KO mice. They also demonstrated that primordial GC apoptosis was increased indicating that Cx43 might be essential for primordial GC survival [28]. To examine the postnatal gonadal development, testes of Cx43 deficient mice were grafted under the kidney capsule of normal adult mice. The reported neonatal GC deficiency persisted postnatally resulting in a “Sertoli-cell-only” (SCO) phenotype. These data lead to the assumption that intercellular communication between SC and GC via Cx43 GJ is also required for postnatal male germ line development [29].

**Figure 1:**
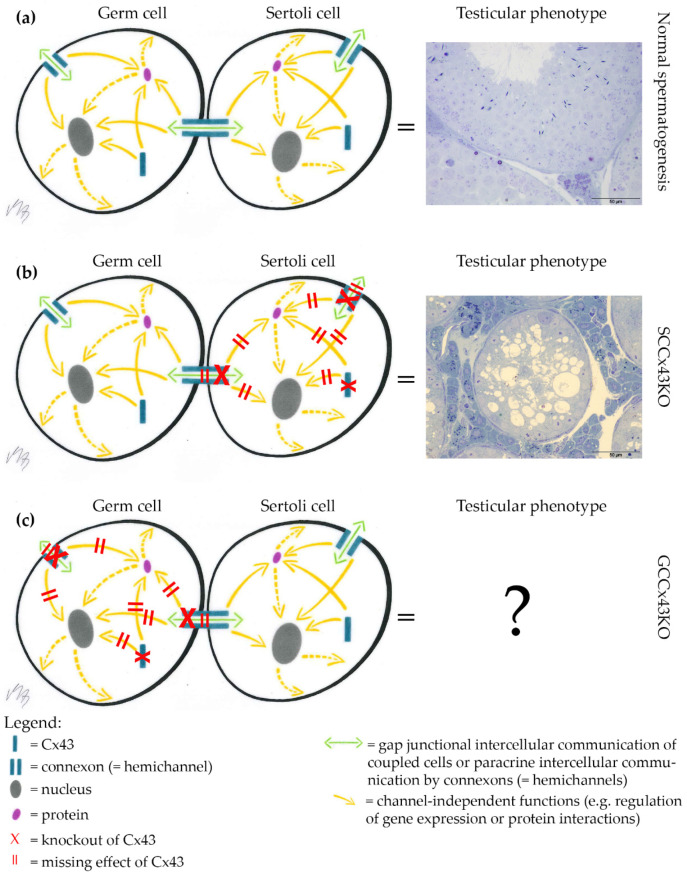
Background of the study. (**a**) Undisturbed intercellular communication between germ cells (GC) and Sertoli cells (SC) via Connexin43 (Cx43)-based gap junctions (GJ) results in normal spermatogenesis. (**b**) Knockout (KO) of Cx43 in SC leads to severely impaired spermatogenesis in SC specific Cx43 KO (SCCx43KO) mice. (**c**) The testicular phenotype of mice, which exhibit a KO of Cx43 in premeiotic or meiotic GC populations, is not confirmed, so far [38]. It is of interest to know, if a GC specific KO of Cx43 leads to a similar phenotype as the KO in SC. Scale bars = 50 µm.

As general Cx43 KO mice are not viable and postnatal testicular development is therefore hard to investigate, our and another working group independently established a conditional KO mouse line using the Cre/loxP recombinase system [30], in which the *Gja1* gene (Gap junction protein alpha 1; coding for Cx43) is knocked out only in somatic SC (SCCx43KO) [31,32]. Male KO mice are viable, but infertile. Using these SCCx43KO mice, the critical role of Cx43 in SC for spermatogenesis has been emphasized. Male KO mice showed reduced testis size and weight, significantly reduced GC numbers, about 95% tubules with arrested spermatogenesis, SCO morphology and/or intratubular cell clusters and consequently infertility [31,32,33]. GJ coupling and Cx43 levels were reduced compared to WT testes and reduced Cx43 levels were associated with, e.g., altered expression of components of the blood-testis barrier [34,35,36]. As shown by various studies, of our research group, the KO of Cx43 in SC has drastic effects on the number, differentiation and gene regulation of GC and consequently also on spermatogenesis [31,37]. Until now, it is not possible to discriminate, if these profound changes of testicular function observed in mice with a general or SC specific KO of Cx43 result from altered communication only between SC or also between SC and GC. Therefore, the question arises, whether there is a similar effect if the *Gja1* gene is knocked out only in GC using efficient and reliable GC specific Cre lines (Figure 1).

A first attempt to elucidate this question was made by Günther et al. (2013), who generated a GC specific KO of Cx43 in mice using transgenic floxed Cx43 and TNAP-Cre mice leading to a Cre-mediated excision of floxed genes in primordial GC [38,39]. In this mouse model, the homozygous KO animals were fertile with normal spermatogenesis and did not show visible alterations of the genital tract, although immunoreactivity of Cx43 protein was decreased and Cx43 mRNA was also downregulated. Expression of other Cxs (Cx26, -45, and -33) was almost unchanged, so the authors concluded that Cx43 did not seem to be essential for GC-SC communication as long as other Cx still existed. However, these data can only be considered as trends as they originate only from a small number of animals. The authors mentioned a high spontaneous and unpredictable mortality rate due to unknown reasons [38]. Furthermore, the actual excision activity of the TNAP-Cre transgenic mouse line is only at around 59.8% and the excision sometimes affects other cells than primordial GC [39]. Therefore, a reason for the missing effect of spermatogenesis might be the insufficient efficiency of Cre activity in the TNAP-Cre mouse line, and ectopic Cre activity in other cells besides primordial GC might have contributed to the increased mortality rate. Both aspects made the TNAP-Cre mouse line insufficient to study the effects of a GC specific deletion of Cx43 further, and more suitable Cre mouse lines should be selected for this purpose. As mentioned above, functional Cx43 based GJs were at least found in spermatogonia and spermatocytes [13,16,21,22], so a Cre line targeting specifically those cell types should be appropriate. The stimulated by retinoic acid gene 8 (*Stra8*)-Cre expression is limited to spermatogonia and preleptotene spermatocytes starting on postnatal day 3 throughout adulthood, before Cx43 protein concentrates at the blood-testis barrier from day 12 onwards in normal mice [35,40]. With an efficiency of >95%, it is considered to be ideal for studying effects of gene loss in those cell types [41,42]. In order to investigate the influence of Cx43-based GJ communication of meiotic GC, the use of protamine-1 (*Prm1*)-Cre might be useful, which starts to be expressed in haploid spermatids and is >92% efficient [42,43].

Thus, aims of the present study were, (1) the establishment of two new conditional KO mouse lines with a GC specific deletion of Cx43 either in premeiotic spermatogonia and early spermatocytes (pGCCx43KO) or in meiotic spermatids (mGCCx43KO) using the Cre/LoxP recombination system to cover the entire postnatal male GC population, (2) investigation of functions of Cx43 in these specific GC populations, and (3) elucidation of possible consequences of the KO of Cx43 in male GC on testicular development and spermatogenesis. Based upon the present data, KO of Cx43 in spermatogonia (pGCCx43KO) or spermatids (mGCCx43KO) did not seem to have dramatic impacts on testicular histology and successful completion of spermatogenesis in adult mice, as it is known for Cx43 in somatic SC [31,32,33,35,36,37,44,45,46]. This might be because GC-Cx43 is not essential for successful spermatogenesis or due to compensation by another, yet not identified, Cx in GC forming heterotypic GJ channels with Cx43 in SC.

## 2. Results

### 2.1. Determination of the Genotype

#### 2.1.1. PCR Genotyping

PCR genotyping using gDNA of mouse ear tissue was performed within the first 14 days of life. Samples were investigated regarding Cre and lox/P site expression. The following genotypes were used for further investigations:

- Cre positive and both alleles of the *Gja1* gene flanked by lox/P sites (“homoflox”) mice were considered as homozygous KO mice
- Cre negative mice were considered as WT mice.

**Figure 2:**
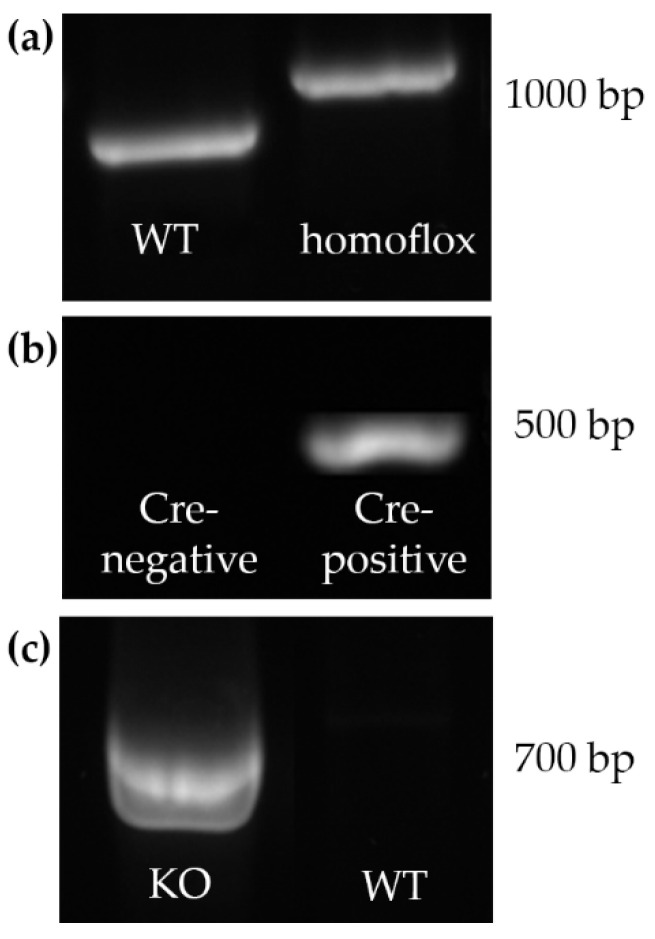
Representative images of PCR genotyping (**a**,**b**) and Connexin (Cx43)-del PCR (**c**). (**a**) Identification of loxP site expression showing no loxP site (=wild type [WT]) and both alleles flanked by loxP sites (“homoflox”); (**b**) Identification of Cre expression with Cre- negative mice showing no band and Cre-positive mice showing a single band at approximately 470 base pairs (bp) (given example was from premeiotic germ cell specific Cx43 knockout (KO) (pGCCx43KO) mice); (**c**) Confirmation of the deletion of Cx43 in testis homogenate was performed by Cx43-del PCR. Primers generated a 670 bp amplicon in KO but not in WT mice (given example was from pGCCx43KO mice).

Representative results are shown in Figure 2a,b.

#### 2.1.2. Cx43-del PCR

To confirm deletion of the floxed *Gja1* alleles in gDNA of testis homogenate, Cx43-del PCR was performed using specific primers generating a 670 bp amplicon of the junction between the intron of Cx43 and the *lacZ* coding region in mice, which lost the Cx43 coding region according to Brehm et al. (2007) [31]. The confirmation of the KO by Cx43-del PCR was successful and representative results are shown in Figure 2c.

### 2.2. Body and Testis Weight

**Figure 3:**
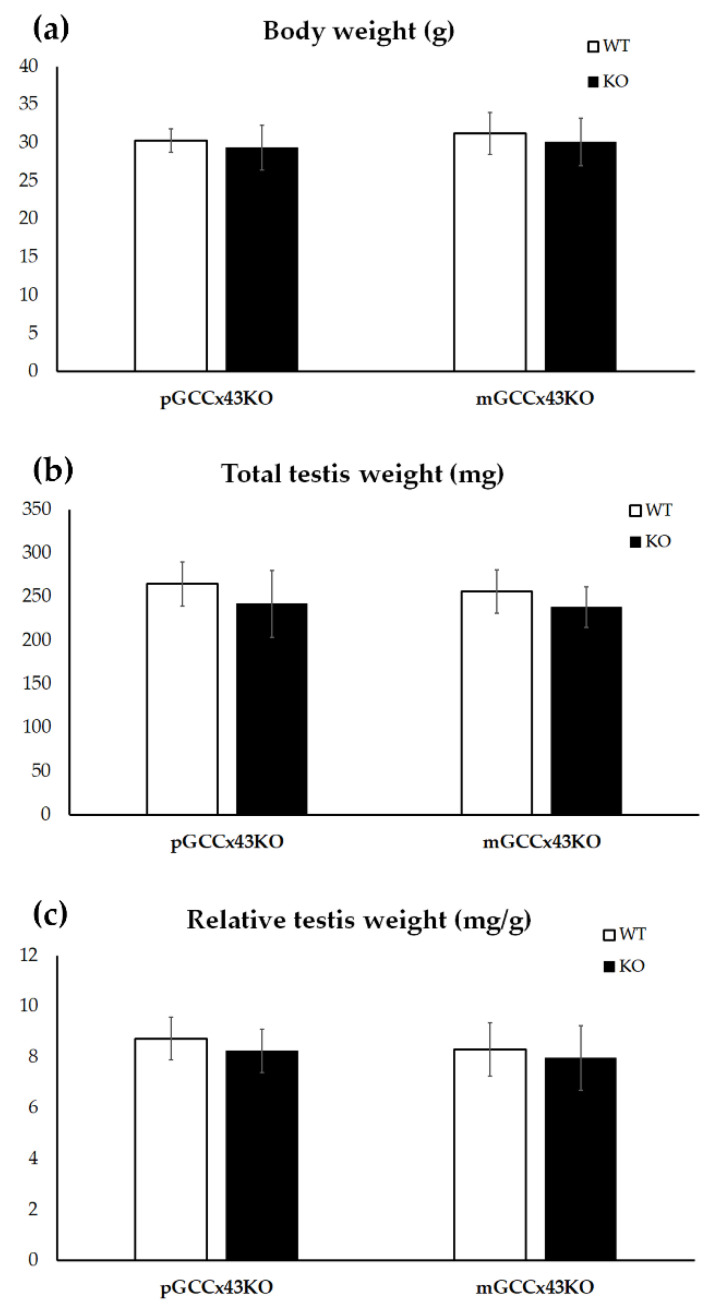
Graphical depiction of the comparison of body weight (**a**), total testis weight (**b**) and relative testis weight (**c**) between knockout (KO) and wild type (WT) mice. No significant differences of the body weight (**a**), the total testis weight (**b**) and the relative testis weight (**c**) could be observed between KO and WT in premeiotic germ cell (GC) specific Connexin43 (Cx43) KO (pGCCx43KO) and meiotic GC specific Cx43 KO (mGCCx43KO) mice. Data are given as mean ± standard deviation.

During dissection of adult male mice, body and total testis weight were determined and relative testis weight was calculated. Mean values were compared between KO and WT. No significant differences could be determined regarding body and total testis weight as well as relative testis weight (Figure 3).

### 2.3. Histology and Immunohistochemistry

#### 2.3.1. Hematoxylin-Eosin (HE) Staining and Tubular Diameter

**Figure 4:**
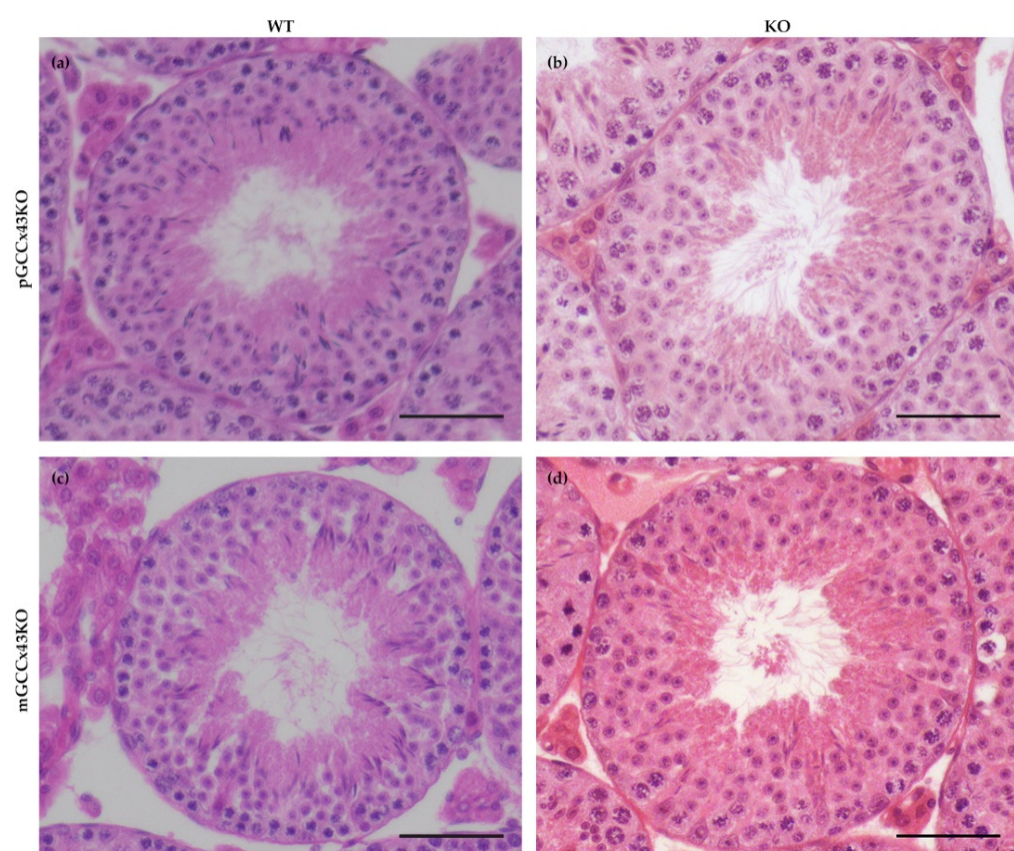
Representative images of a seminiferous tubule of adult wild type (WT) (**a**,**c**) and knockout (KO) (**b**,**d**) premeiotic germ cell (GC) specific KO of Connexin43 (Cx43) (pGCCx43KO; (**a**,**b**)) and meiotic GC specific KO of Cx43 (mGCCx43KO; **c**,**d**) mice. No obvious differences in testicular morphology and spermatogenesis were visible between KO and WT, when Cx43 was knocked out in either premeiotic (**a**,**b**) or meiotic (**c**,**d**) GC. Hematoxylin eosin staining; scale bars = 50 µm.

**Figure 5:**
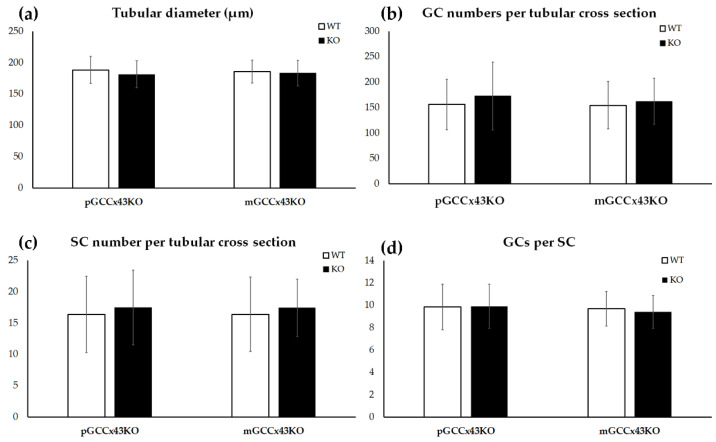
Graphical depiction of the comparison of the tubular diameter (**a**), germ cell (GC) numbers per tubular cross section (**b**), Sertoli cell (SC) numbers per tubular cross section (**c**) and calculated GC/SC ratio per tubular cross section (**d**) between knockout (KO) and wild type (WT) mice. No significant differences in any of the examined parameters could be observed between KO and WT in premeiotic GC specific KO of Connexin43 (Cx43) (pGCCx43KO) and meiotic GC specific KO of Cx43 (mGCCx43KO) mice. Data are given as mean ± standard deviation.

In HE stained sections, no obvious alterations of spermatogenesis in adult KO male mice were visible compared to corresponding WT littermates (Figure 4). Furthermore, the mean diameter of seminiferous tubules did not differ significantly comparing adult KO and WT animals of both mouse lines, respectively (Figure 5). Original data can be found in Supplemental Table S1 and Supplemental Table S2.

#### 2.3.2. Cx43 Localization

**Figure 6:**
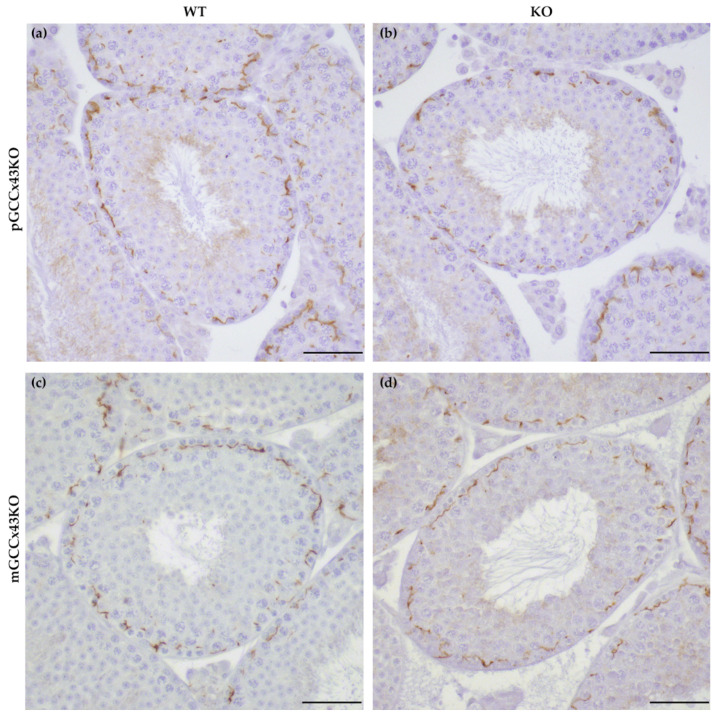
Immunohistochemical detection of Connexin43 (Cx43) protein in adult testes of wild type (WT; (**a**,**c**)) and knockout (KO; (**b**,**d**)) mice of the premeiotic germ cell (GC) specific KO of Cx43 (pGCCx43KO; (**a**,**b**)) and meiotic GC specific Cx43 KO (mGCCx43KO; (**c**,**d**)) mouse line. No obvious differences were visible between KO and WT, when Cx43 was knocked out in either premeiotic (**a**,**b**) or meiotic (**c**,**d**) GC. Scale bars = 50 µm.

Specific immunoreaction of Cx43 protein was found in the basal area of the seminiferous epithelium of adult WT and KO mice of both GCCx43KO mouse lines using IHC. No obvious differences between WT and KO male mice could be observed. As other cell types remain unaffected by the deletion of Cx43, this staining pattern probably represents Cx43 GJ between adjacent SC at the level of the BTB (Figure 6).

**Figure 7:**
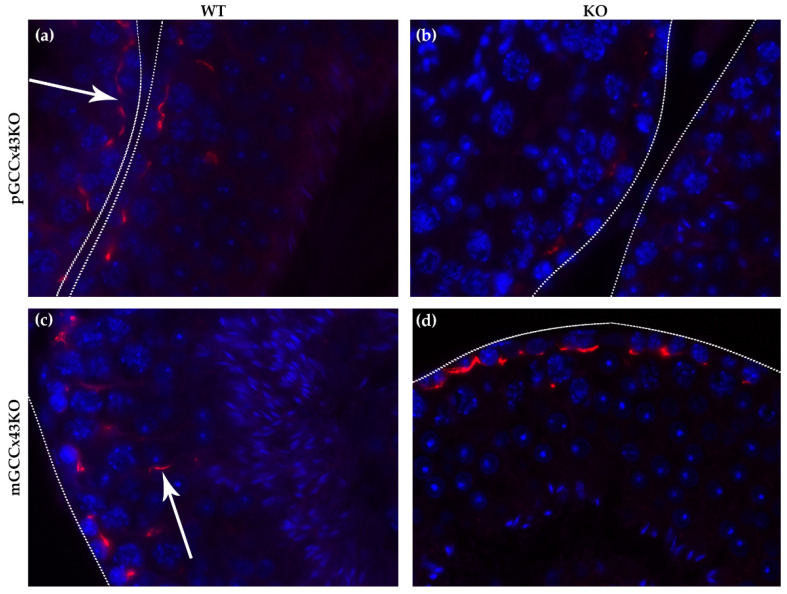
Representative localization of Connexin43 (Cx43) in adult p- and mGCCx43KO and WT mice. For easier orientation, the basal lamina of the tubules is marked by dotted lines. Cx43 forms a fine linear staining pattern (red) in the basal area of the seminiferous tubules (arrow in (**a**)) in WT mice of both mouse lines (**a**,**c**) resulting from synthesis by both Sertoli cells and basally located germ cells. Furthermore, Cx43 can also be found more apically in the seminiferous epithelium (arrow in (**c**)) in WT mice. In the KO animals of the pGCCx43KO mouse line (**b**), the described staining pattern in the basal area of the seminiferous epithelium is also visible; however, the staining intensity seems to be less intense, probably resulting from the lack of Cx43 in the basally located germ cells due to the KO in spermatogonia/early spermatocytes. In the mGCCx43KO mouse line (**d**), the apical localization of Cx43 could not be detected indicating that the more apically localized germ cells (spermatids) do not synthesize Cx43 following its KO in those cell types. Magnification: 630×.

Using more sensitive immunofluorescence (IF) analysis of Cx43 localization, it was visible that Cx43 forms a fine linear staining pattern in the basal area of the seminiferous tubules in WT mice of both mouse lines resulting from synthesis by both Sertoli cells and basally located germ cells (Figure 7, arrow in a). Furthermore, Cx43 can also be found more apically in the seminiferous epithelium (Figure 7, arrow in c) in WT mice. In the KO animals of the pGCCx43KO mouse line (Figure 7b), the described staining pattern in the basal area of the seminiferous epithelium is also visible, however, the staining intensity seems to be less intense, probably resulting from the lack of Cx43 in the basally located germ cells due to the KO in premeiotic GC. In the mGCCx43KO mouse line (Figure 6d), the apical localization of Cx43 could not be detected (Figure 7d) indicating that the more apically localized germ cells (spermatids) may not synthesize Cx43 following its KO in meiotic GC.

Furthermore, using immunogold labeling and subsequent transmission electron microscopy (TEM), the loss of Cx43 could also be confirmed (Supplemental Figures S2–S5).

#### 2.3.3. Sox9-Staining and GC and SC Numbers

Numbers of SC and GC per tubular cross section were determined in immunohistochemically stained testicular sections using the primary anti-Sox9 antibody, which represents an SC marker and allows an accurate distinction of SC and GC (Supplemental Figure S1). In adult mice of both GCCx43KO mouse lines, SC and GC numbers did not differ significantly between genotypes and consequently, also the GC:SC ratio was not significantly different (Figure 5).

### 2.4. Semi-Quantitative Western Blot Analysis

**Figure 8:**
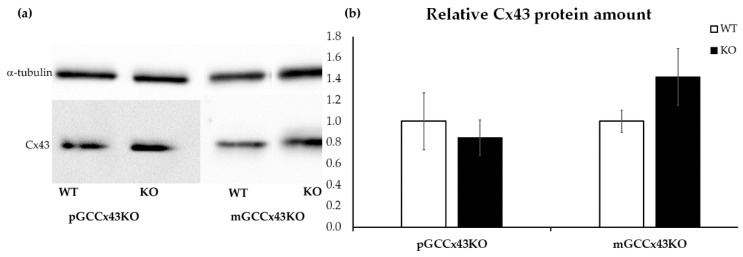
Representative Western blot of Connexin43 (Cx43) (43 kilodalton [kDa]) (**a**) and the corresponding semi-quantitative evaluation by densitometry (**b**) using testis homogenate. α-tubulin (52 kDa) was used as loading control (housekeeper). No significant differences in the relative protein amount of Cx43 could be determined comparing knockout (KO) and wild type (WT) mice of both mouse lines.

The semi-quantitative WB analysis of three adult mice per genotype of each mouse line with α-tubulin as a loading control showed no significant differences in the amount of Cx43 protein in testis homogenate of KO and WT mice (Figure 8).

### 2.5. qRT-PCR

**Figure 9:**
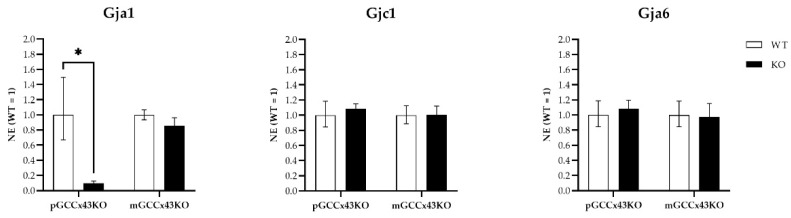
qRT-PCR of gap junction protein alpha 1 (*Gja1*; coding for Connexin43 [Cx43]), gap junction protein gamma 1 (*Gjc1*; coding for Cx45) and gap junction protein alpha 6 (*Gja6*; coding for Cx33). mRNA of *Gja1* was significantly decreased (* *p* < 0.05) following the knockout (KO) of Cx43 in adult premeiotic germ cell (GC) specific KO of C43 (pGCCx43KO) mice, while it was only slightly reduced in meiotic GC specific KO of Cx43 (mGCCx43KO) mice. *Gjc1* and *Gja6* mRNA did not differ significantly between genotypes in both p- and mGCCx43KO mice. Data are given as geometric mean ± geometric standard deviation.

To quantify mRNA expression, qRT-PCR of different Cx family members known to be expressed in GC, namely *Gja1* (coding for Cx43), *Gjc1* (gap junction protein gamma 1; coding for Cx45), and *Gja6* (gap junction protein alpha 6; coding for Cx33) was performed. *Gja1* expression was significantly downregulated in pGCCx43KO mice (Figure 9), which was an expected finding due to its deletion in premeiotic GC. However, in mGCCx43KO mice, *Gja1* was also reduced, yet not statistically significant, maybe because the amount of Cx43 mRNA in meiotic GC does not contribute significantly to the total amount of Cx43 mRNA in whole testis homogenate. To identify possible compensatory increases of other Cx types expressed in GC, mRNA expression of Cx33 and Cx45 was also investigated, but no significant differences could be observed comparing KO and WT testes of both p- and mGCCx43KO mice (Figure 9). Original data can be found in Supplemental Table S3.

### 2.6. Mating Experiments

Fertility assessment of homozygous KO males of both mouse lines revealed no obvious disturbance of mating behavior or fertility. Male KO mice were able to produce offspring within six weeks with an average litter size of six pups.

## 3. Discussion

Several studies have shown that Cx43 is essential for spermatogenesis [27,28,29,31,32,33,34,35,36,37,44,45,46,47]. However, the contribution of known hemichannels provided by GC to this important role could not be determined and consequently is unknown so far. With the present study, we addressed this issue by creating GC specific KO of the predominant testicular GJ protein Cx43. Two new conditional KO mouse lines lacking the GJ protein Cx43 in different GC populations have successfully been generated using the Cre/loxP recombination system. In pGCCx43KO mice, Cx43 was knocked out in premeiotic spermatogonia and early spermatocytes using *Stra8*-Cre mice, whereas in mGCCx43KO mice, *Gja1* was deleted in meiotic spermatids (using *Prm1*-Cre mice) to study the role of Cx43 based GJIC in those cell types. Using qRT-PCR, an expected reduction of *Gja1* following its deletion was confirmed. The decrease of *Gja1* mRNA was statistically significant in pGCCx43KO mice concomitant with previous results [38], while only a decreasing trend could be observed in mGCCx43KO mice (Figure 9). The observation that Cx43 mRNA was only slightly reduced in mGCCx43KO might be explained by the amount of Cx43 mRNA in meiotic GC (spermatids), which possibly does not contribute significantly to the total amount of Cx43 mRNA in whole testis homogenate [10,11,13,22]. At protein level, no significant changes of the amount of Cx43 could be observed using semi-quantitative WB analysis (Figure 8), and protein localization seemed to be unchanged comparing KO and WT animals of both mouse lines using IHC (Figure 6). However, using more sensitive methods like IF (Figure 7) and immunogold electron microscopy (Supplemental Figures S2–S5), the lack of Cx43 in either premeiotic (pGCCx43KO) or meiotic GC (mGCCx43KO) could be demonstrated. The discrepancy between mRNA and protein amount of Cx43 might originate from the different accuracy of semi-quantitative WB analysis and qRT-PCR, of which the latter is more reliable than semi-quantitative WB analysis. Data of the present study regarding Cx43 protein synthesis might indicate that Cx43 in premeiotic or meiotic GC only constitutes a small (non-significant) part of the total amount of Cx43, whereas deletion of Cx43 in SC results in a significant downregulation of both Cx43 protein synthesis and mRNA expression [18,31]. Thus, the major proportion of Cx43 protein in the testis seems to be provided by SC and Leydig cells [14,15,21].

Unlike the KO of Cx43 in SC [31,32,33,35,45,46], its deletion in spermatogonia/early spermatocytes or spermatids did not have dramatic impacts on testicular histology and successful completion of spermatogenesis in adult p- and mGCCx43KO mice (Figure 4 and Figure 7) confirming previous results of Günther and colleagues [38]. These authors also generated a GC specific KO of Cx43 using TNAP-Cre mice leading to a Cre-mediated excision of floxed Cx43 in primordial GC [39]. Homozygous KO animals were also fertile with normal spermatogenesis. In this study, expression of other Cx was almost unchanged, so the authors concluded that Cx43 did not seem to be essential for GC-SC communication if other Cxs still existed [38]. Similar results were obtained in the present study by investigating mRNA expression of other Cxs, Cx33 and Cx45, which are known to be expressed in GC [11,13,14,15,16,21,22,48,49] and might be elevated to compensate for the loss of Cx43 in GC by forming heterotypic GJ channels with SC. Cx45 and Cx33 can interact with Cx43 [50,51], and thus might be able to balance out the loss of Cx43 in GC. In the present study, mRNA expression of *Gjc1* (coding for Cx45) and *Gja6* (coding for Cx33) did not show any significant differences between WT and KO animals in both p- and mGCCx43KO mouse lines, indicating that these two Cx members probably do not compensate for the loss of Cx43. Our results, together with those of Günther et al. [38], indicate growing evidence that Cx43 in GC might not play an essential role in regulating spermatogenesis. So far, implications that another Cx might compensate for the loss of Cx43 in GC are missing. The present results suggest that either SC-GC crosstalk via homotypic Cx43-based GJ is not necessary for normal termination of spermatogenesis, or that GC-Cx43 is replaced by another, yet not identified, Cx, resulting in heterotypic GJ channels. It might also be speculated that, while homotypic Cx43 based GJIC between SC is essential for spermatogenesis [31,32,33,34,35,36,37,44,45,46], SC-GC communication might normally occur via heterotypic GJ composed of Cx43-hemichannels supplied by SC, and connexons of GC assembled by a so far unidentified Cx type. If this was the case, SC-GC communication would not be affected by a GC specific Cx43 KO, but an SC specific deletion of Cx43 would impede the communication between those cell types.

Functional differences of SC-SC vs. SC-GC GJ have already been described. While GJIC can occur efficiently and bi-directionally between adjacent SC, it takes place only uni-directionally from SC to spermatogonia and late spermatocytes in rats [16,22]. Furthermore, it has been described that SC-SC coupling is nonselective, whereas GJ connection between SC and spermatogonia is strongly selective for positively charged biotin tracers compared to negatively charged Lucifer Yellow [22]. Differences in gating properties of GJ are associated with their constituting proteins [52]. It was therefore proposed that the Cxs of those selective SC-spermatogonia junctions might differ from SC-SC junctions by the connection via heterotypic channels [22]. Our results, together with findings in SCCx43KO mice, can support this suggestion as the KO of Cx43 in SC has drastic effects on testicular function, while its KO in GC does not lead to obvious alterations of spermatogenesis and fertility. It was also described that dye coupling between SC and adluminal spermatids was too weak to be detected by fluorescence microscopy, which supports our results of the Cx43 KO in meiotic GC [22]. However, the molecular composition of SC-GC junctions remains unknown.

**Figure 10:**
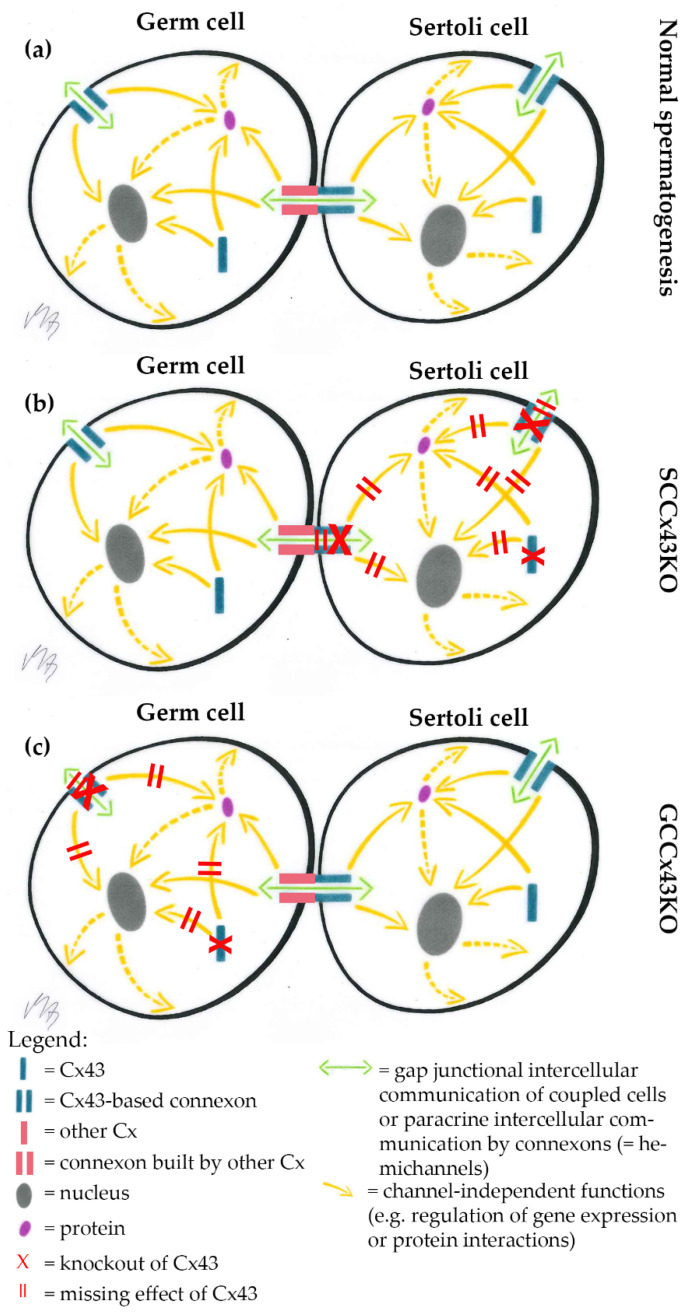
Hypothetical explanatory model of heterotypic Sertoli cell (SC)-germ cell (GC) gap junctional intercellular communication (GJIC). (**a**) In wild type (WT) mice, GJIC is undisturbed resulting in normal spermatogenesis; (**b**) SC specific knockout (KO) of Cx43 leads to impaired GJIC between SC and GC as the Connexin43 (Cx43)-based hemichannel of the heterotypic gap junction channel is missing in SC specific Cx43 KO (SCCx43KO) mice; (**c**) GJIC via heterotypic hemichannels remains unaffected, when Cx43 is deleted in premeiotic or meiotic GC (GCCx43KO).

Previous studies of our research group using SCCx43KO mice showed that deletion of the *Gja1* gene in SC affects gene expression in prepubertal mice and, surprisingly, the majority of the significantly regulated genes were GC specific and involved in the regulation of the onset of meiosis [37,44]. This indicates a close connection between SC and GC and that Cx43 in SC participates in the regulation of GC gene expression, GC proliferation and differentiation [37,44]. However, it might also be possible that these changes were caused by missing channel-independent functions of Cx43. These involve the direct exchange of substances between the cytosol and the extracellular space includin, e.g., the participation in the release of ATP and NAD^+^, Ca^2+^ wave propagation, and the passage of survival signals within the tissue via hemichannels. There is also evidence that even Cxs alone can display functions of intracellular signaling, independently from GJ formation (e.g., influence on directional cell migration and cell polarity, interactions with the cytoskeleton) mainly mediated by the cytoplasmic C-terminus [5,6,7,28,53,54,55,56]. However, there is evidence that small interfering RNAs can be transported via GJ to modulate gene expression in the target cell [57,58,59,60,61], and some findings in SCCx43KO were replicated by adding GJ blockers to an SC line in vitro [34], thus, it might be hypothesized that many effects of the deletion of Cx43 observed in SCCx43KO mice are channel-dependent. Conversely, there is evidence that also GCs regulate SC function and that this influence depends on the GC type and its particular requirements, but little is known about the role of GJ/Cx43 in this process [61,62,63,64,65,66,67]. It has been shown in vitro that GCs influence the protein synthesis of several tight junction proteins built by SC [61]. Similar results were shown by O’Shaughnessy et al. (2008), who investigated several SC specific mRNA transcripts after GC depletion in mice. Their results indicated that GC participate in regulating SC activity [66]. However, the mechanisms by which GC regulate SC activity are not clearly understood so far. Su et al. (2013) proposed a possible way to influence SC function by GC could be through transmitting small RNAs (microRNAs, small interfering RNAs, piwi-interacting RNAs) via GJ channels [61]. Recently, it has been shown in invertebrate testes that bi-directional GJ communication between soma and germ line is required for proper spermatogenesis [68,69]. All these data support a possible role for GJ in GC in regulating spermatogenesis also in vertebrates. However, the present results of the newly generated p- and mGCCx43KO mice indicate that Cx43 in spermatogonia/early spermatocytes or spermatids is not the key player in GC for these processes in mice. Still, heterotypic GJ, with Cx43-hemichannels from SC and GC-hemichannels built up by another Cx type, might be involved in the regulation of spermatogenesis (Figure 10). This model might be able to explain, why an SC specific KO of Cx43 results in such drastic effect on spermatogenesis and primarily affects GC [31,33,34,35,36,37,44,45,46], while the KO of Cx43 does not seem to compromise spermatogenesis in mice with a GC specific KO of Cx43 [38].

Finally, the present results further underline the established idea that somatic SC and their Cx43-based GJ are crucial for GC development. Based upon the data of the present study and previous finding in SCCx43KO mice [18,31,32,33,34,35,36,37,44,45,46], we hypothesize that SC and GC possibly communicate via heterotypic GJ composed of one Cx43-based SC-connexon and a GC-hemichannel constituted of a so far unknown Cx family member. Consequently, future investigations should focus on the exact composition of heterocellular GJ, linking SC and GC to identify possible binding partners in GC for Cx43-connexons of SC. A GC specific deletion of a potential binding partner in GC for SC-Cx43 hemichannels could elucidate, if there is another Cx member in GC, which can resemble the testicular phenotype of SCCx43KO mice.

## 4. Materials and Methods

### 4.1. Animals

Mice lacking the Cx43 gene (*Gja1*) solely in specific GC generations were generated using the Cre/loxP recombinase system [31,32]. This tool allows the generation of conditional mutant mice lacking a certain gene specifically in a targeted organ or cell type. The Cre (causes recombination)/loxP (locus of crossing over) recombinase system originates from the bacteriophage P1 and ensures an excision of a gene sequence flanked by two loxP-sites (so called “floxed” gene sequences) by the Cre recombinase [30]. It is based on mating two transgenic mouse lines, one of them having the target gene flanked by loxP sites, the other expressing the Cre recombinase under a cell/tissue specific promotor. Briefly, homozygous Cx43-floxed mice (*Gja1* gene flanked by loxP sites) were mated with either *Stra8*-Cre mice (for Cx43 KO in premeiotic GC, pGCCx43KO mice) or *Prm1*-Cre mice (for Cx43 KO in meiotic GC, mGCCx43KO mice). Offspring (F1 generation: heterozyously Cx43-floxed and Cre positive) were then backcrossed with homozygously Cx43-floxed mice resulting in homozygously Cx43-floxed and either Cre-positive (considered as KO) or Cre-negative (considered as WT) offspring. All animal experiments were conducted according to the German Animal Protection Law and approved by the Lower Saxony State Office for Consumer Protection and Food Safety (decision 33.19-42502-04-17/2513, July 2017).

### 4.2. PCR Genotyping

Determination of the genotype was performed within the first 14 days *post natum* (p.n.) using ear tissue during the marking process of the animals. Genomic DNA was extracted from the tissue using Direct PCR tail reagent (VWR Life Science, catalogue no. 732-3256) according to the manufacturer’s protocol and was analyzed for the expression of loxP-sites (Cx43-flox) and the Cre enzyme under specific promotors (primers are shown in Table 1). To analyze loxP-site expression, PCR conditions were set according to Brehm et al. (2007). Cre enzyme expression was investigated using the following PCR conditions; briefly, 1 µL of gDNA was added to 5 µL of 5× Green GoTaq^®^ Flexi buffer (Promega, Mannheim, Germany), 2 µL of MgCl2 (25 mmol/L; Promega), 0.5 µL of dNTPs (Promega), 0.5 µL of GoTaq^®^ Flexi DNA polymerase (Promega), 0.5 µL of each primer (10 µmol/L; Eurofins Genomics, Ebersberg, Germany), and diethylpyrocarbonate (DEPC)-H_2_O to a final volume of 25 µL. Cycler programs were as follows for the amplification of Cre in the pGCCx43KO mouse line: 1 × 95 °C for 2 min; 30 × [95 °C for 1 min, 58 °C for 2 min, and 72 °C for 2 min], 72 °C for 7 min. For the Cre detection in mGCCx43KO mice, PCR conditions were as follows: 1 × 95 °C for 2 min; 35 × [95 °C for 2 min, 60 °C for 30 s, and 72 °C for 45 s], 72 °C for 7 min. PCR products were separated in 1% agarose gels and visualized with GelRed^®^ (Biotrend, Köln, Germany).

### 4.3. Confirmation of the KO via Cx43-Del PCR

Deletion of the *Gja1* gene in testicular tissue was confirmed by Cx43-del PCR using gDNA from testes homogenate as previously described by Brehm and colleagues [31]. Primers are shown in Table 1.

### 4.4. Tissue Sampling and Treatment

Adult (>60 days) male mice from both mouse lines were sacrificed by cervical dislocation after anesthesia with CO_2_. Testes were surgically removed and either placed into Bouin’s solution for 48 h or snap frozen in liquid nitrogen and stored at −80 °C until further processing. Bouin fixed tissue samples were embedded in paraffin and 4 µm thick testicular sections were placed onto glass slides for either hematoxylin eosin (HE) staining or IHC/IF.

### 4.5. Body and Testis Weights

During dissection, body and total testis weight were determined and relative testis weight was calculated; per genotype 11 male mice were analyzed of the pGCCx43KO mouse line and five male mice per genotype of the mGCCx43KO mouse line.

### 4.6. Testicular Histology

For investigation of the testicular phenotype, HE stained testicular sections were analyzed via light microscopy (Zeiss Axioscope, Jena, Germany; Olympus DP 70 camera and Olympus DP Soft V3.2 software, Olympus Hamburg, Germany). Mean diameter of seminiferous tubules was determined of ten randomly selected round tubular cross sections in Bouin-fixed and HE-stained sections of three different adult KO and WT siblings of both KO mouse lines each.

### 4.7. Immunohistochemistry and Immunofluorescence

To visualize Cx43 localization in KO and WT testes, IHC was performed using a specific antibody. Furthermore, the SC-specific marker Sox9 was used to accurately distinguish SC from GC and to determine their numbers. Antibodies, their dilutions and applications are shown in Table 2.

For IHC, Bouin-fixed and paraffin-embedded testicular 4 µm section mounted on glass slides (Histobond, Paul Marienfeld, Laboratory Glassware, Lauda-Königshofen, Germany) were used. During deparaffinization and rehydration, the sections were incubated at room temperature in 196 mL 80% alcohol containing 4 mL of 30% hydrogen peroxide for 30 min to block endogenous peroxidase activity, followed by heat-induced antigen retrieval in sodium citrate buffer (pH 6) at 96–99 °C for 20 min. Afterwards, sections were blocked with phosphate-buffered saline (PBS) containing 3% of bovine serum albumin (BSA) at room temperature for 20 min and incubated with the corresponding primary antibody at 4 °C overnight in a humidified chamber. Sections were then exposed to the compatible secondary antibody EnVision+ Single Reagent (HRP. Rabbit) for 30 min and immunoreactivity was visualized by the diaminobenzidine (DAB) detection system. After counterstaining with haematoxylin, sections were dehydrated and covered with Eukitt^®^ (O. Kindler GmbH, Freiburg, Germany). For negative controls, the primary antibody was omitted and replaced by PBS.

Numbers of SC and GC per tubular cross section and GC:SC ratio were determined in immunohistochemically stained testicular sections using the primary anti-Sox9 antibody. Per genotype, 45 randomly chosen round tubular cross sections of three different animals were analyzed and mean values were calculated.

For IF, Bouin-fixed and paraffin embedded testicular sections were deparaffinized and rehydrated, pretreated using citric buffer (pH 6) for 20 min at 96–99 °C and blocked using 3% BSA (in PBS) for 20 min at room temperature. The sections were incubated overnight with the primary antibody (see Table 2) at 4 °C. The next day, sections were exposed to the fluorophore Alexa 546 (see Table 2) for 45 min at room temperature and mounted using Prolong^®^ Gold Antifade Reagent Antifade (Invitrogen, Darmstadt, Germany, P36930). The sections were viewed under a Zeiss Axiovert 200M fluorescence microscope (Carl Zeiss, Oberkochen, Germany, Zeiss Axiovert 200M).

### 4.8. Semi-Quantitative WB Analysis

In order to confirm the specificity of antibodies used for IHC and to investigate whether relative protein amount of Cx43 was reduced in p- and mGCCx43KO testes, semi-quantitative WB analysis using testes homogenate of three mice per genotype was performed. To ensure that equal amounts of protein were investigated, α-tubulin was used as a loading control. Antibodies and their dilutions used for WB analysis are shown in Table 2.

Proteins were extracted from testicular tissue using TRIzol^®^ reagent following the manufacturer’s instructions and were fractionated by SDS-PAGE (sodium dodecyl sulfate polyacrylamide gel electrophoresis) in a polyacrylamide gel at 150 V for 80 min with an amount of 20 µg protein per lane. The PageRuler^TM^ Prestained Protein Ladder 10–170 kDa (Fermentas, St. Leon-Rot, Germany) was used as a marker to determine molecular weight. Fractionated proteins were then blotted onto a Protran BA 85 nitrocellulose membrane (Whatman, Dassel, Germany) for 60 min at 1 A/cm^2^ (PeqLab, Erlangen, Germany). Afterwards, the membrane was blocked with 5% non-fat skimmed milk in Tris-buffered saline and tween 20 (TBS-T) for 60 min, followed by incubation with the primary antibody for Cx43 diluted in Tris buffered saline (TBS) over night at 4 °C. The next day, the secondary goat-anti rabbit IgG-horseradish peroxidase (HRP) antibody was diluted 1:5000 in TBS and was applied for 45 min before the membrane was treated with the SuperSignal^®^ West Dura Kit (Thermo Scientific, Schwerte, Germany) following the manufacturer’s protocol. Finally, the membrane was photographed on a Bio 1D, Vilber (Lourmat, Eberhardzell, Germany). After that, the membrane was incubated twice in in stripping buffer for 30 min and the membrane was then incubated solely in TBS for the negative control at 4 °C over night. The next day, incubation with the secondary antibody, treatment with SuperSignal^®^ West Dura Kit (Thermo Scientific, Schwerte, Germany), photographing and stripping of the membrane was repeated before the primary antibody of the loading control α-tubulin was applied at 4 °C over night. Secondary antibody incubation, visualization and photography were repeated once more.

A semi quantitative analysis of Cx43 protein relative to the housekeeper (loading control) α-tubulin was performed using the Bio-1D advanced software (Vilber Lourmat, Germany).

### 4.9. qRT-PCR

RNA extraction, reverse transcription and quantification gene of interest expression were performed as described in Schenke et al. [71] from testis samples of four mice per genotype with three technical raplicates each. Relative gene expression levels were calculated according to the 2^∆∆CT^ method [72]. Mean of the Ct values of the reference genes *Actb* and *Hsp90ab1* was used for reference gene normalization for each sample as previously described [38,44,45].

To test for successful DNAse digestion, cDNA samples were subjected to a *Sox9* PCR. *Sox9* encodes for a SC specific transcription factor and the primers (see Table 1) used for the digest test are located on two different exons, so if the cDNA sample was contaminated with gDNA, the amplicon would be 810 bp, while the cDNA amplicon would only be 201 bp.

### 4.10. Mating Experiments

In order to test whether male m- and pGCCx43KO mice were fertile, homozygous KO of both mouse lines were mated with WT females (Janvier, C57BL/6JR) for up to six weeks.

### 4.11. Statistical Analyses

Significance was determined using a student’s *t*-test or a Welch’s test for samples with unequal variances for the comparison of tubular diameter, intratubular cell numbers, semi-quantitative WB analysis, and qRT-PCR. A *p*-value of <0.05 was defined as significant with * *p* < 0.05.

## Figures and Tables

**Table 1:** Primers used for PCR genotyping, Connexin43 (Cx43)-del PCR and qRT-PCR.

Name	Forward Primer (5′-3′)	Reverse Primer (5′-3′)	Size of Amplicon	Objective	Reference
Cx43-flox	TCATGCCCGGCACAAGTGAGAC	TCACCCCAAGCTGACTCAACCG	1100 bp (flox) 988 bp (WT)	Genotyping	[31]
Stra-8 Cre	TCTGATGAAGTCAGGAAGAACC	GAGATGTCCTTCACTCTGATTC	470 bp	Genotyping	
Prm1-Cre	AGGCAAATTTTGGTGTACGG	GTTCCCTCAGCAGCATTCTC	405 bp	Genotyping	
Cx43-del	GGCATACAGACCCTTGGACTCC	TGCGGGCCTCTTCGCTATTACG	670 bp	Cx43-del PCR	[31]
*Gja1* (Cx43)	ACAGCGGTTGAGTCAGCTTG	GAGAGATGGGGAAGGACTTGT	106 bp	qRT-PCR	[37]
*Gjc1* (Cx45) NM_001001496.2	CAGTTCTGGTGAACAGGGCA	ACAATCAGCACAGTGAGCCA	125 bp	qRT-PCR	
*Gja6* (Cx33) NM_001159382.1	GCCAAGCTGCAAGAGTAGGA	CAAGTGAGTGCACACCTGAG	138 bp	qRT-PCR	
*Actb* NM_007393.5	CGCAGCCACTGTCGAGTC	GTCATCCATGGCGAACTGGT	96 bp	qRT-PCR	
*Hsp90ab1* NM_008302.3	GCTCCTTCGCTATCACACCT	TTGCTCTTTGCTCTCACCAGT	121 bp	qRT-PCR	
*Sox9*	CGGAGGAAGTCGGTGAAGA	GTCGGTTTTGGGAGTGGTG	810 bp (gDNA) 201 bp (cDNA)	DNAse digest test	[70]

**Table 2:** Antibodies used for immunohistochemistry (IHC) and Western blot (WB) analysis.

Antibody	Host	Application	Dilution	Company	Catalogue No.
Anti-Sox9	Rabbit	IHC	1:800	Merck Millipore	AB5535
Cx43	Rabbit	IHC IF	1:500	Cell Signaling	3512
		WB	1:1000		
α-Tubulin	Rabbit	WB	1:1000	Cell Signaling	2125
2nd Anti-Rabbit	Goat	WB	1:5000	Santa Cruz	SC-2004
EnVision+ Single Reagent (HRP Rabbit)	Goat	IHC	ready-to-use	Agilent Dako	K400311-2
2nd Alexa 546		IF	1:1000	Invitrogen	A11010

## Data Availability

The data presented in this study are available in Supplemental Tables S1–S3.

## Supplementary Materials

The following are available online at https://www.mdpi.com/article/10.3390/ijms22157924/s1.
