# Supplementary material for: Connexin43 in Germ Cells Seems to Be Dispensable for Murine Spermatogenesis
Source: Int J Mol Sci. 2021 Jul 25;22(15):7924. doi: 10.3390/ijms22157924 (PMC8348783; doi:10.3390/ijms22157924)
Supplement: Supplementary file 1 [file ijms-22-07924-s001.zip › Figures S2-S5.pdf]

Supplemental figures S2-S5:

For more accurate localization of Connexin43 (Cx43), immunogold labeling using 10 nm gold particles on Bouin-fixed and paraffin-embedded testicular sections for transmission electron microscopy (TEM) was performed. Afterwards, the sections were transferred onto an epoxy resin pin and prepared for ultra-thin sections.
Analyzing the samples via TEM, immunogold particles could be detected in both Sertoli cells and rarer in germ cells in wild type (WT) animals of both mouse lines (Supplemental Figure S2, Supplemental Figure S3). Comparing these samples to the correspondent knockout (KO) samples, it was visible that Cx43 could not be detected in basally located germ cells in KO animals of the pGCCx43KO mouse line, but still in Sertoli cells (Supplemental Figure S4). Accordingly, in KO animals of the mGCCx43KO mouse line, Cx43 was missing in apically located spermatids (Supplemental Figure S5). With these results, we provide evidence that the KO of Cx43 in early and late germ cell populations using Cre/LoxP recombinase system probably was successful and support our hypothesis that Cx43 in those specific germ cells is not essential for spermatogenesis and male fertility.

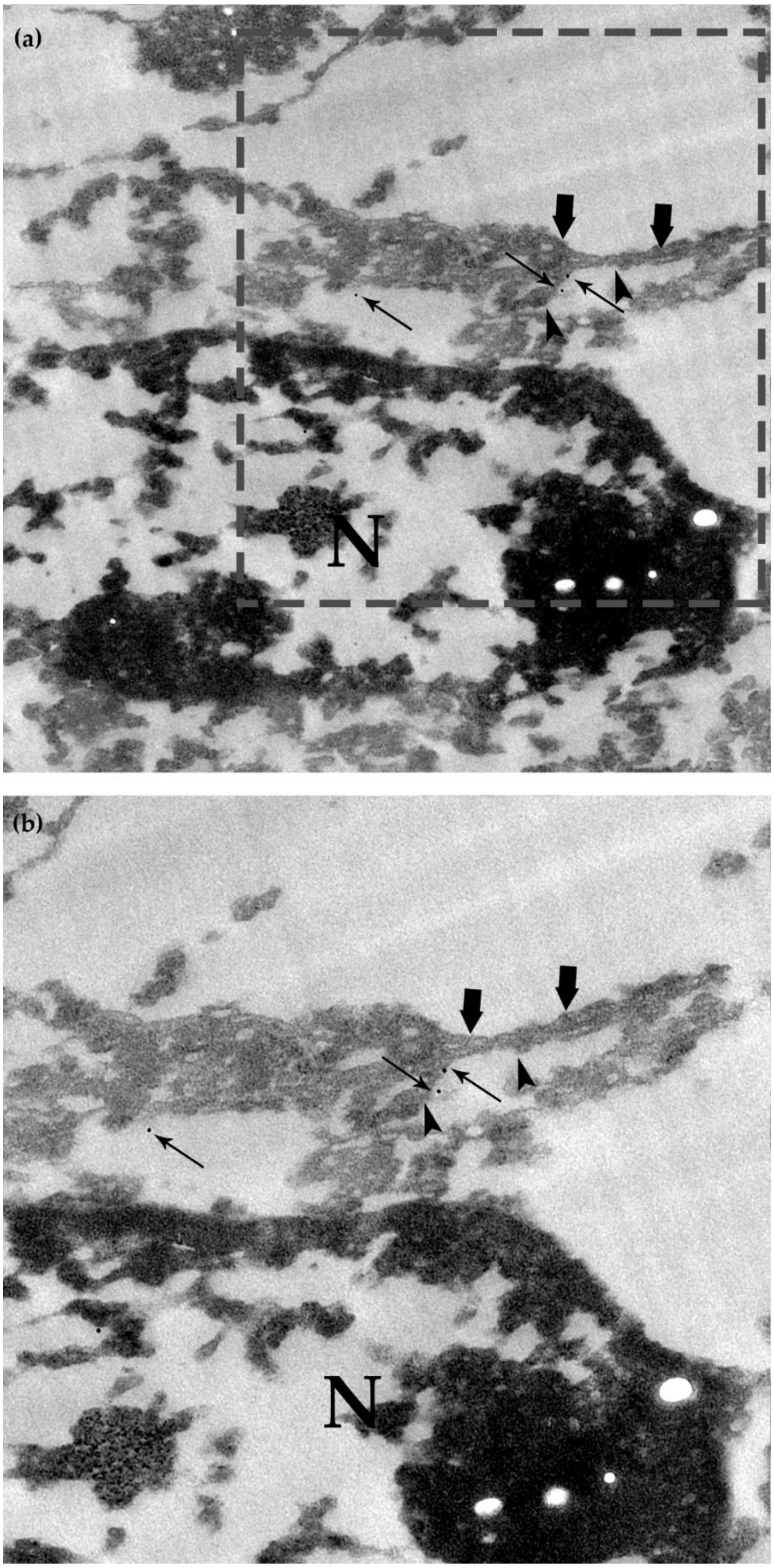

Supplemental Figure S1: Representative pictures of transmission electron microscopy with Connexin43 (Cx43) immunogold labeling in a wild type (WT) animal of the pGCCx43KO mouse line. Immunogold particles (marked by thin arrows) were found in both Sertoli cells (not shown in the pictures) and basally located germ cells. Arrowheads pointing towards the germ cell membrane, while bold arrows mark the Sertoli cell membrane. The gray box in (a) marks the region depicted in (b). N marks a nucleus of a spermatogonium. Magnification: (a) 12500x; (b) 20000x.

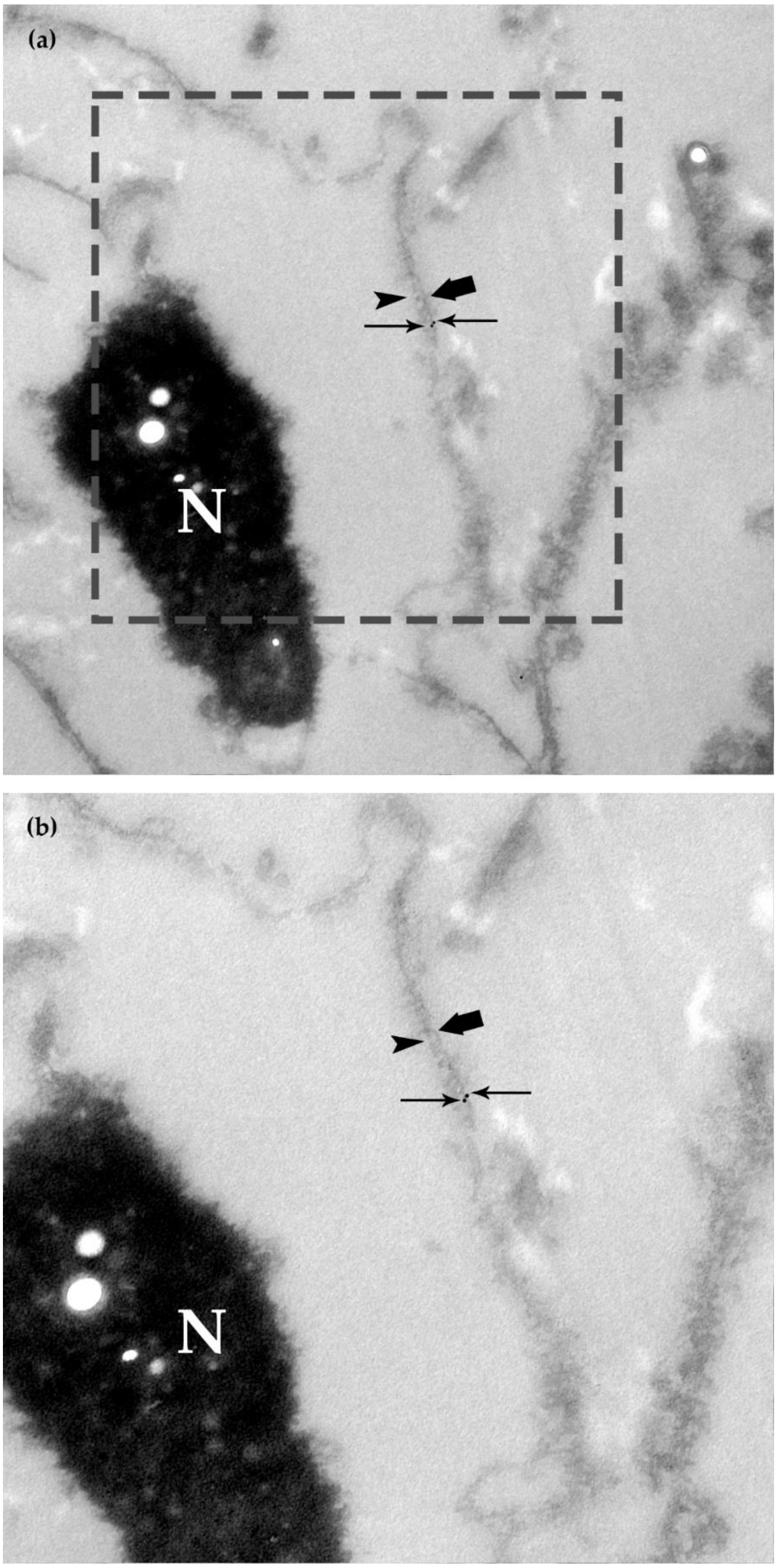

Supplemental Figure S2: Representative pictures of transmission electron microscopy with Connexin43 (Cx43) immunogold labeling in a wild type (WT) animal of the mGCCx43KO mouse line. Immunogold particles (marked by thin arrows) were found in both Sertoli cells and apically located germ cells. Arrowheads pointing towards the germ cell membrane, while bold arrows mark the Sertoli cell membrane. The gray box in (a) marks the region depicted in (b). N marks a nucleus of a spermatid. Magnification: (a) 12500x; (b) 20000x.

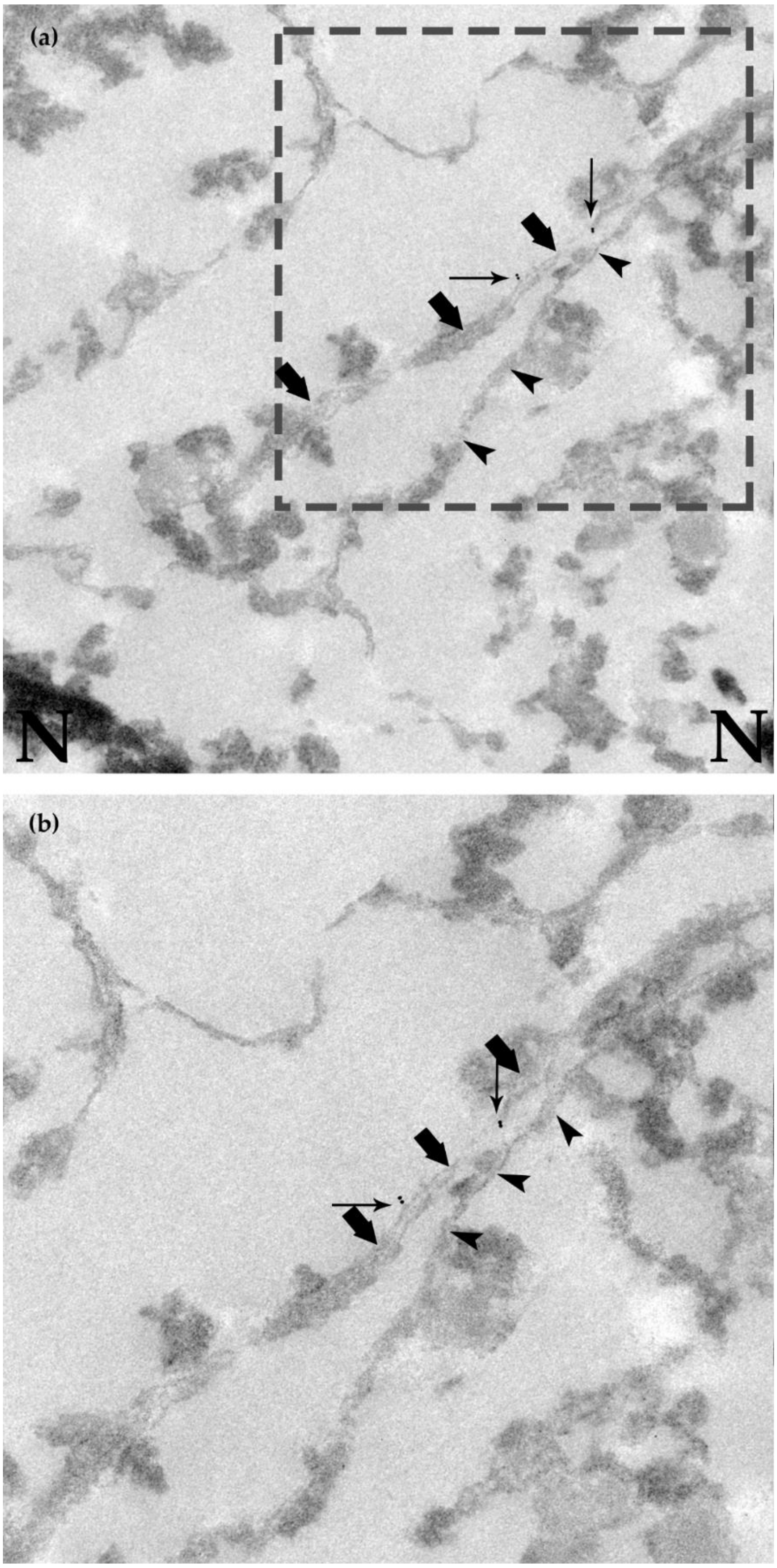

Supplemental Figure S3: Representative pictures of transmission electron microscopy with Connexin43 (Cx43) immunogold labeling in a knockout (KO) animal of the pGCCx43KO mouse line. Immunogold particles (marked by thin arrows) were only found in Sertoli cells, but not in basally located germ cells. Arrowheads pointing towards the germ cell membrane, while bold arrows mark the Sertoli cell membrane. The gray box in (a) marks the region depicted in (b). N marks a nucleus of a spermatogonium. Magnification: (a) 12500x; (b) 20000x.

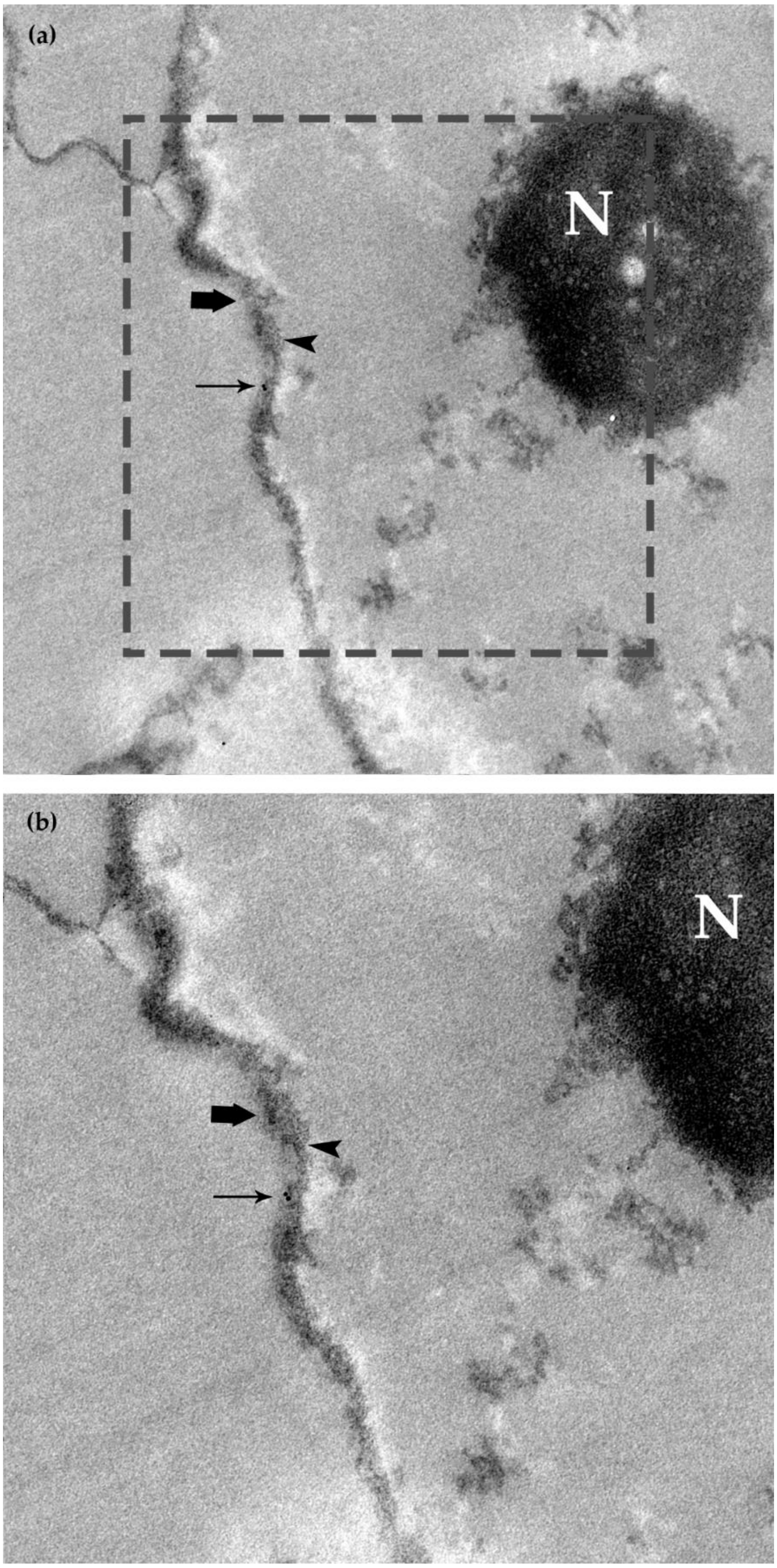

Supplemental Figure S4: Representative pictures of transmission electron microscopy with Connexin43 (Cx43) immunogold labeling in a knockout (KO) animal of the mGCCx43KO mouse line. Immunogold particles (marked by thin arrows) were only found in Sertoli cells, but not in apically located germ cells. Arrowheads pointing towards the germ cell membrane, while bold arrows mark the Sertoli cell membrane. The gray box in (a) marks the region depicted in (b).). N marks a nucleus of a round spermatid. Magnification: (a) 12500x; (b) 20000x.
