# Supplementary material for: Connexin43 in Germ Cells Seems to Be Dispensable for Murine Spermatogenesis
Source: Int J Mol Sci. 2021 Jul 25;22(15):7924. doi: 10.3390/ijms22157924 (PMC8348783; doi:10.3390/ijms22157924)
Supplement: Supplementary file 1 [file ijms-22-07924-s001.zip › Supplemental Figure S1.pdf]

Supplemental Figure S1:

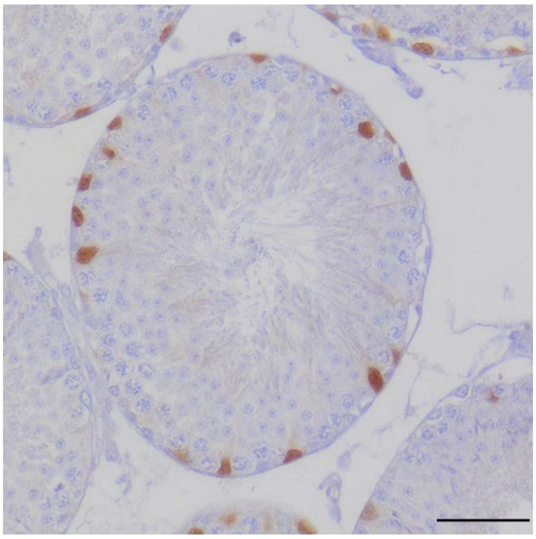

Figure S1: Example for a Sox9 immunohistochemistry. Sox9 is a marker for Sertoli cell nuclei, which allows accurate distinction between Sertoli cell nuclei (brown) and germ cell nuclei, which are counterstained with hematoxylin. Scale bar = 50 µm.
