# Supplementary material for: Connexin43 in Germ Cells Seems to Be Dispensable for Murine Spermatogenesis
Source: Int J Mol Sci. 2021 Jul 25;22(15):7924. doi: 10.3390/ijms22157924 (PMC8348783; doi:10.3390/ijms22157924)
Supplement: Supplementary file 1 [file ijms-22-07924-s001.zip › Supplemental_Table S1_pGCCx43KO.pdf]

pGCCx43KO

Bodyweight	
KO	WT
31.2	32.41
31.73	25.43
28.74	28.18
25.52	26.14
28.91	31.19
29.09	30.28
30.37	27.32
29.38	31.16
29.42	32.11
28.59	34.43
30.07	34.09

Total Testis weight (mg)	
KO	WT
230.00	250.00
230.00	180.00
200.00	260.00
200.00	210.00
230.00	310.00
280.00	280.00
250.00	270.00
260.00	280.00
260.00	260.00
270.00	310.00
250.00	300.00

Relative Testis weight (mg/g)	
KO	WT
7.37	7.71
7.25	7.08
6.96	9.23
7.84	8.03
7.96	9.94
9.63	9.25
8.23	9.88
8.85	8.99
8.84	8.10
9.44	9.00
8.31	8.80

Cell Counts:

WT				KO		
GC	SC	GC/SC		GC	SC	GC/SC
189	23	8.2		249	24	10.4
368	45	8.2		192	18	10.7
202	23	8.8		203	20	10.2
104	16	6.5		242	22	11.0
106	13	8.2		163	18	9.1
110	9	12.2		160	15	10.7
115	12	9.6		205	20	10.3
72	8	9.0		231	24	9.6
116	10	11.6		261	26	10.0
118	13	9.1		188	18	10.4
126	10	12.6		88	9	9.8
124	15	8.3		142	15	9.5
104	11	9.5		81	12	6.8
72	12	6.0		129	10	12.9
99	11	9.0		66	15	4.4
162	18	9.0		99	9	11.0
206	21	9.8		83	10	8.3
152	18	8.4		124	12	10.3
198	23	8.6		119	13	9.2
150	17	8.8		110	13	8.5
191	21	9.1		149	9	16.6
157	14	11.2		146	14	10.4
226	25	9.0		164	19	8.6
187	19	9.8		107	11	9.7
159	19	8.4		125	14	8.9
141	14	10.1		121	18	6.7
158	10	15.8		101	15	6.7
142	12	11.8		89	15	5.9
152	21	7.2		130	17	7.6
178	16	11.1		100	9	11.1
158	14	11.3		174	18	9.7
170	17	10.0		235	23	10.2
139	13	10.7		313	28	11.2
127	10	12.7		337	35	9.6
127	12	10.6		297	30	9.9
170	15	11.3		256	25	10.2
169	19	8.9		238	21	11.3
163	22	7.4		241	22	11.0
178	19	9.4		260	24	10.8
118	14	8.4		222	23	9.7

222	14	15.9		164	14	11.7
178	15	11.9		144	15	9.6
228	18	12.7		190	14	13.6
173	20	8.7		180	16	11.3
126	15	8.4		149	14	10.6

WB - Densitometry

	WT			KO		
Tubulin	78274529	51104676	87866450	54156689	60101734	74734244
Cx43	3961621	3796015	5919905	2915769	4297291	9267590
	19.7582073	13.4627171	14.8425439	18.5737241	13.9859586	8.06404297
