# Supplementary material for: Connexin43 in Germ Cells Seems to Be Dispensable for Murine Spermatogenesis
Source: Int J Mol Sci. 2021 Jul 25;22(15):7924. doi: 10.3390/ijms22157924 (PMC8348783; doi:10.3390/ijms22157924)
Supplement: Supplementary file 1 [file ijms-22-07924-s001.zip › Supplemental_Table S2_mGCCx43KO.pdf]

mGCCx43KO

Bodyweight	
**KO**	**WT**
26.03	27.05
32.44	32.97
27.77	34.58
31.08	27.86
33.28	33.61

Total Testis weight (mg)	
**KO**	**WT**
210.00	290.00
210.00	230.00
270.00	270.00
240.00	230.00
260.00	260.00

Relative Testis weight (mg/g)	
**KO**	**WT**
8.06761429	10.7208872
6.47348952	6.97603882
9.72272236	7.80798149
7.72200772	8.25556353
7.8125	7.73579292

Cell Counts:

WT				KO		
GC	SC	GC/SC		GC	SC	GC/SC
173	20	8.7		135	17	7.9
143	17	8.4		166	18	9.2
181	20	9.1		189	23	8.2
198	22	9.0		188	19	9.9
168	19	8.8		179	23	7.8
173	20	8.7		147	19	7.7
143	17	8.4		185	21	8.8
181	20	9.1		306	29	10.6
198	22	9.0		192	16	12.0
168	19	8.8		101	13	7.8
136	17	8.0		146	15	9.7
92	13	7.1		166	19	8.7
98	10	9.8		121	11	11.0

156	17	9.2		153	12	12.8
128	11	11.6		88	12	7.3
173	19	9.1		224	26	8.6
192	18	10.7		217	23	9.4
203	23	8.8		158	19	8.3
204	19	10.7		172	20	8.6
365	45	8.1		237	24	9.9
182	22	8.3		211	25	8.4
141	12	11.8		122	11	11.1
129	15	8.6		165	19	8.7
133	10	13.3		123	13	9.5
134	12	11.2		99	14	7.1
123	14	8.8		136	17	8.0
77	9	8.6		98	10	9.8
106	13	8.2		142	16	8.9
130	17	7.6		160	16	10.0
95	14	6.8		156	17	9.2
197	21	9.4		143	13	11.0
176	18	9.8		156	12	13.0
198	18	11.0		112	13	8.6
164	16	10.3		187	17	11.0
183	17	10.8		105	16	6.6
162	15	10.8		121	13	9.3
160	15	10.7		113	10	11.3
167	18	9.3		83	11	7.5
112	8	14.0		224	20	11.2
145	13	11.2		201	22	9.1
83	8	10.4		173	21	8.2
133	12	11.1		210	21	10.0
128	13	9.8		190	18	10.6
102	10	10.2		206	20	10.3
119	9	13.2		196	19	10.3

WB - Densitometry

	**WT**			**KO**		
Tubulin	73929247	61382268	68376621	66636051	68270591	58167364
Cx43	25679383	21667680	19292989	16540428	12384994	16326295
	2.87893393	2.83289526	3.54411755	4.02867755	5.51236367	3.56280246
